# Dielectric Signatures of Late Carcinoma Immune Cells Using MMTV-PyMT Mammary Carcinoma Models

**DOI:** 10.1021/acsomega.4c04210

**Published:** 2024-09-26

**Authors:** Raphael Oladokun, Christopher Smith, Timothy Eubank, Soumya Srivastava

**Affiliations:** † Department of Chemical & Biomedical Engineering, West Virginia University, Morgantown, West Virginia 26506-6201, United States; ‡ Department of Microbiology, Immunology & Cell Biology, West Virginia University, Morgantown, West Virginia 26506-6201, United States

## Abstract

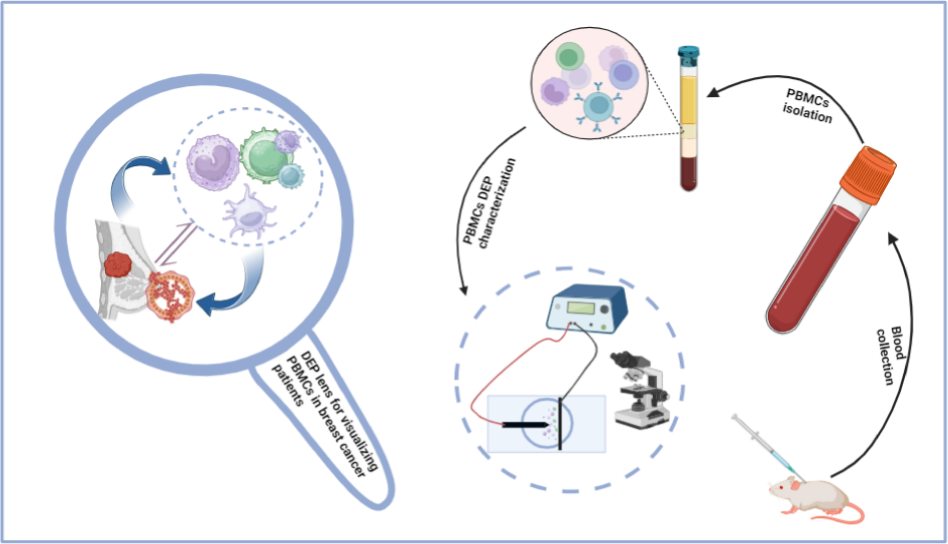

Peripheral blood mononuclear cells (PBMCs) are specialized immune cells produced from hematopoietic stem cells (HSC). They actively surveil for any signs of infection, foreign invaders, and abnormal or aberrant cells associated with diseases. Numerous inherent interactions between PBMCs and proliferating cancer cells facilitate cellular communication, inducing alterations in the composition of the PBMCs. These subtle alterations can be detected by using dielectrophoresis (DEP). The ultimate objective is to apply this knowledge in a clinical setting to achieve noninvasive early detection of breast cancer while minimizing the occurrence of false positives and negatives commonly associated with standard screening methods like mammography. To realize our long-term goal, we are probing the dielectric properties of the PBMCs from FVB/N MMTV-PyMT+ (late carcinoma, PyMT+ PBMC) and FVB/N (wild-type, WT-PBMC) age-matched mice at 14+ weeks using dielectrophoresis on a microfluidic platform. The central hypothesis of this research is that the changes triggered in the subcellular components, such as the cytoskeleton, lipid bilayer membrane, cytoplasm, focal adhesion proteins, and extracellular matrix (ECM) at the onset of carcinoma, regulate dielectric properties (conductivity, σ; and permittivity, ε), thus affecting the bioelectric signals that aid in the detection of breast cancer. The ANOVA results suggest a significant difference in PyMT+ PBMCs crossover frequencies at 0.01 and 0.05 S/m medium conductivity levels. Post hoc pairwise analysis of WT-PBMCs showed that the crossover frequencies are distinct across the medium conductivity ranges from 0.01 to 0.05 S/m. This study revealed that on average, PyMT+ PBMCs have increased crossover frequency, polarizability, higher membrane capacitance, and a folding factor compared with the age-matched wild-type PBMCs.

## 1 Introduction

Breast cancer (BC) is a complex and heterogeneous disease. In 2023, the American Cancer Society revealed that an estimated 297,790 new cases of invasive breast cancer are expected to be diagnosed in U.S. women, along with 43,170 deaths in females.^1^ The progression depends on tumor-host cell interaction.^2^ It has become a major public health concern due to its high prevalence and the heterogeneity of characteristics present in genomics events, gene expression, metastatic potential, and treatment response.^3^ This type of cancer occurs when mammary cells undergo uncontrolled proliferation, invade nearby tissues, and promote metastases. In mice, the mouse mammary tumor virus (MMTV) is acknowledged as the primary etiological agent of mammary tumors. Identifying MMTV-like sequences and antigens in human mammary carcinoma has supported the theory that a virus homologous to MMTV (namely, HMTV) may be involved in human breast cancer.^2^ Understanding breast cancer and its molecular mechanisms is crucial and has been greatly aided by using transgenic mouse models.^4^ These models allow for manipulating genetic events frequently observed in breast cancers. Genetically engineered mouse models (GEMMs) specifically target mammary tissue to induce tumorigenesis through tissue-specific genetic manipulation.^4^ Over the years, various GEMMs have been developed, each with unique characteristics and genetic manipulations that mimic aberrant signaling events found in human breast cancers.^5^ These models have played a crucial role in unraveling the molecular events involved in breast cancer initiation, progression, and metastasis^5^ and have emerged as a clinically relevant and invaluable tool for cancer researchers.^6^ The mammary-specific polyomavirus middle T antigen overexpression mouse model (MMTV-PyMT), first published in 1992, is the most commonly used genetically engineered mouse model (GEMM) for cancer research. The mammary lesions in Mouse Mammary Tumor Virus-Polyoma Middle T antigen (MMTV-PyMT) mice closely mirror the molecular and histological progression observed in human breast tumors, making it a valuable tool for cancer researchers and instrumental in understanding tumor biology.^5^ This model, known for its rapid development of multifocal tumors and extensive lung metastasis, remains the most widely used GEMM in cancer research.^5^ Several available transgenic breast cancer mouse models undergo spontaneous mammary tumorigenesis upon genetic manipulation that models aberrant signaling events observed in human breast cancers.

The mammary tumors developed in MMTV-PyMT breast cancer mice mainly go through four stereotypical stages, including hyperplasia at 4 to 6 weeks of age, adenoma/mammary intraepithelial neoplasia at 8–9 weeks of age, early malignant between 8 and 12 weeks of age and late-malignancy after 13 weeks.^7,8^ It is important to note that MMTV has been extensively studied as a model for understanding breast cancer and viral oncogenesis. While the virus is primarily associated with mouse mammary tumors, evidence suggests a potential link between MMTV and breast cancer in humans. However, the significance of MMTV in human breast cancer remains a subject of ongoing research and debate within the scientific community.^9,10^ On the other hand, MMTV WT (wild type) is the nonmutated, naturally occurring form of the mouse mammary tumor virus (MMTV). It is a reference or control when studying the effects of mutated or modified virus versions.^7^ WT is where the MMTV-PyMT Tg (transgenic) is not bred into the animal, i.e., not genetically crossed.

This study aims to use the model to detect the late malignancy stage using dielectrophoresis (DEP). The long-term goal is to build a noninvasive diagnostic tool for screening breast cancers at an early stage. To achieve this, peripheral blood mononuclear cells (PBMCs) were obtained from both Polyoma middle T antigen-driven breast cancer mice and wild-type (WT) models at stage IV (14+ weeks). We introduce a novel approach using DEP, an electrokinetic technique, that effectively addresses significant limitations in characterizing PBMCs from MMTV-PyMT+ and WT mice. Dielectrophoresis is a noninvasive technique that allows for the characterization and analysis of bioparticles, particularly biological cells. We hypothesize that the dielectric properties of these cells, such as membrane capacitance, conductance, conductivity, and cytoplasm conductivity, are different and unique based on the cell phenotype and genotype. We aim to quantify and compare these properties to elucidate the disparities between WT and MMTV-PyMT+ (late-stage) mice. This study represents the first instance in which dielectrophoresis is employed in this context, showcasing its potential to overcome various drawbacks associated with traditional methods.

## 2 Theory

H. A. Pohl initially described dielectrophoresis, where he investigated its efficacy in removing suspended solid particles from polymer solutions.^11^ Nascimento et al. (2008) later defined dielectrophoresis as a technique for manipulating dielectric particles such as DNA, proteins, and cells.^12^ Understanding the passive electrical properties, such as dielectric constant and conductivity, of different cellular components like the cell membrane, cytoplasm, and nucleus contributes to a better understanding of cell functions.^13^ These electrical properties reflect the cell’s ability to maintain ion balances and indicate metabolic work and biological organization. Fundamental dielectric properties of a cell include cell membrane capacitance, cytoplasmic conductance, permittivity, and membrane resistance. The motion of suspended particles relative to the surrounding medium is driven by the polarization forces generated by an inhomogeneous (nonuniform) electric field. The equations in our previous studies established a theoretical description of the time-averaged DEP force on a spherical particle in conventional dielectrophoresis as a function of the frequency of the applied electric field.^14−17^

### 2.1 Single Shell Model

The frequency-dependent dielectric properties of biological cells can be described using a single-shell model, which is particularly suitable when the crossover frequency is well below 1 MHz.^18−20^ Crossover frequency measurements involve determining the frequency at which the dielectrophoretic force acting on the cells becomes zero, resulting in no movement of the cells. The single-shell model simplifies the cell as a homogeneous spherical particle. In cases where the crossover frequency is significantly lower than 1 MHz, the conductivity of the cell’s interior is much greater than that of the plasma membrane. As a result, the approximate expression for the specific membrane capacitance can be  and conductance .^18,21−24^ The lower or first crossover frequency can be expressed in terms of the capacitance and conductance as given below:
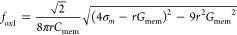
1where δ is the membrane thickness, *r* is the cell radius, *σ*_int_ is the cell interior conductivity, *σ*_m_ is the membrane conductivity, and *f*_*xo*1_ is the lower (first) crossover frequency. This relationship can be simplified further to the form shown in eq 2 for G_mem_ ≤ 600 S/m^2^, given by^25^
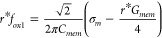
2

## 3 Materials and Methods

For the isolation of peripheral blood mononuclear cells (PBMCs), the following reagents were used: 1X PBS (Gibco), Ficoll-Paque Plus (density = 1.077g/mL, GE Healthcare 17–1440–03), 1X RBC Lysis Buffer (Invitrogen eBiosciences 00–4333–57), RPMI-1640 media (Gibco), and Heparin sodium (liquid, NDC 25 021–400–10).

The procedure for PBMC isolation was conducted as follows. Mice were humanely sacrificed using airway isoflurane in a sealed chamber, and euthanasia was confirmed by opening the peritoneal cavity. A 19-gauge bore needle filled with heparin sodium solution was inserted into the right atrium to collect approximately 1 mL of whole blood. The collected blood was transferred into a 15 mL conical tube and mixed with an equal volume of 1X PBS by vortexing. In a new 15 mL conical tube, 4 mL of Ficoll-Paque medium was added, and the whole blood was gently layered on top without mixing. Centrifugation was performed at 2,000 rpm for 20 min at room temperature with the brake OFF, separating the sample into layers. The top serum layer was removed, leaving the cotton-like “buffy cell” layer (PBMCs). The buffy layer was collected and transferred to a 50 mL conical tube. The tube was filled to 40 mL with RPMI-1640 media and centrifuged at 1,000 rpm for 8 min at 4 °C with the brake ON. The media was then completely aspirated without disturbing the cell pellet. The pellet was resuspended in 5 mL of prewarmed RBC lysis buffer and incubated in the dark for 4 min at room temperature. Following the incubation, the lysis reaction was stopped by adding 30 mL of 1X PBS. The tube was centrifuged at 1,000 rpm for 8 min at 4 °C, and the media was aspirated without disturbing the cell pellet. Finally, the pellet was resuspended in 300 μL of PBS for further experiments and analysis.

The isolated cells were re-suspended in an isotonic solution media with a low conductivity (280 mOs/kg). To prepare this solution media, 8.6 g of Fisher Sucrose, 0.3 g of anhydrous VWR Dextrose, and 0.1 g of Sigma-Aldrich Bovine Serum Albumin (BSA) were dissolved in 100 mL of deionized water and vigorously stirred. The conductivity of the medium was adjusted by using Lonza Minimum Essential Medium Eagle (EMEM) media to achieve the desired conductivity. By adding 1 mL of Eagle’s media to 40 mL of the prepared media, a medium conductivity of 0.04 S/m at pH 7.4 was obtained. This ratio could be adjusted to achieve the desired conductivity. For the experiments, five conductivity buffers (media) were used: 0.01 0.02, 0.03, 0.04, and 0.05 S/m.

**Figure 1:**
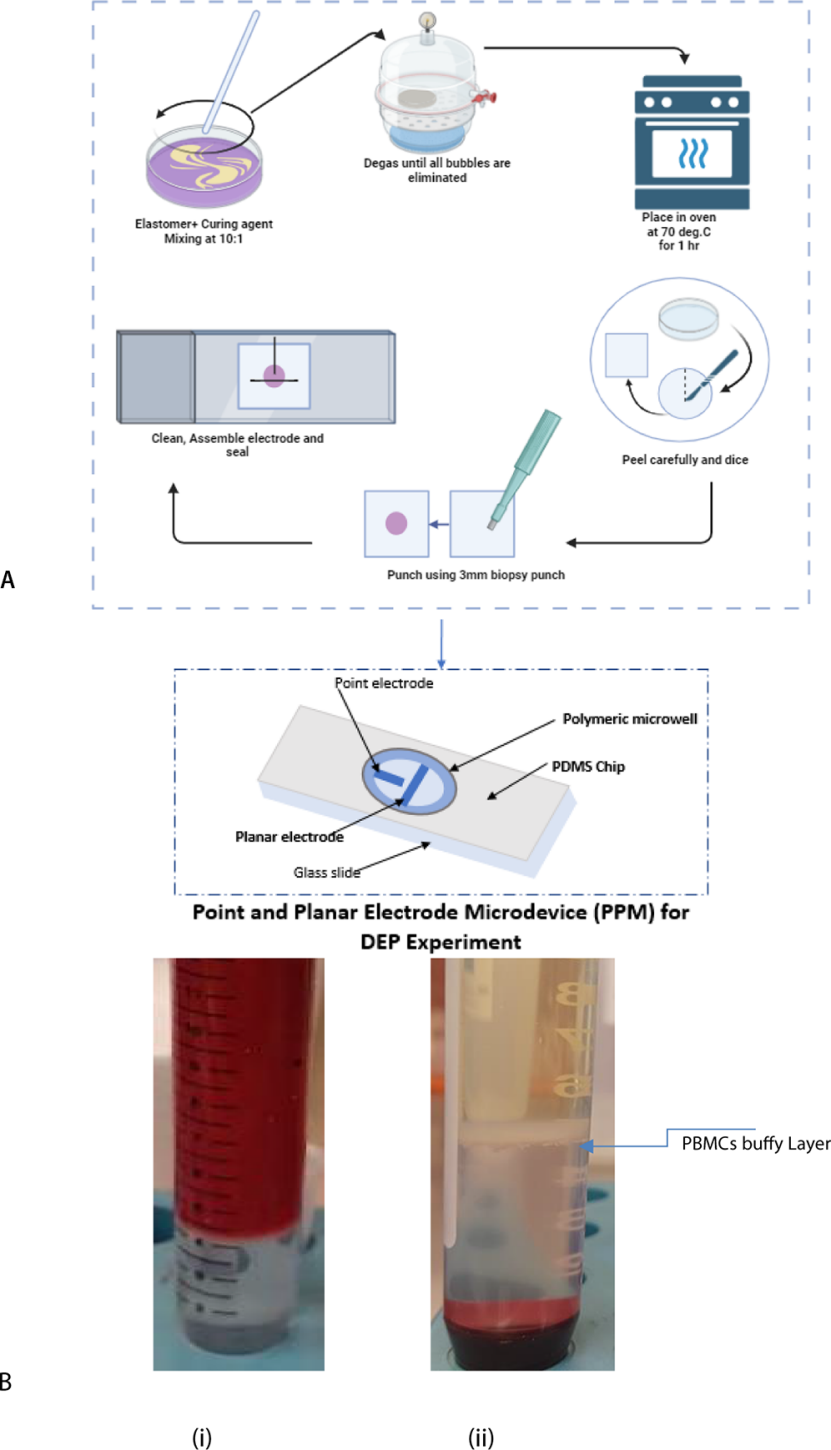
(A) Block diagram of the experimental procedure for fabricating a PDMS (poly(dimethylsiloxane)) PPM (point-and-planar microwell) device.^19,30^ (B) (i) A mixture of PBS and whole blood was layered over the Ficoll medium before centrifugation. (ii) Blood samples separated into RBC, plasma, medium, and PBMCs.

For fabrication, as described in our previous work,^26−29^ rapid prototyping was used to fabricate the point-and-planar electrode microwell device. A block diagram illustrating this process is depicted in Figure 1A, and the separation of PBMC cells from whole blood is shown in Figure 1B, indicated by the label *PBMCs buffy layer*.

## 4 Results and Discussion

**Figure 2:**
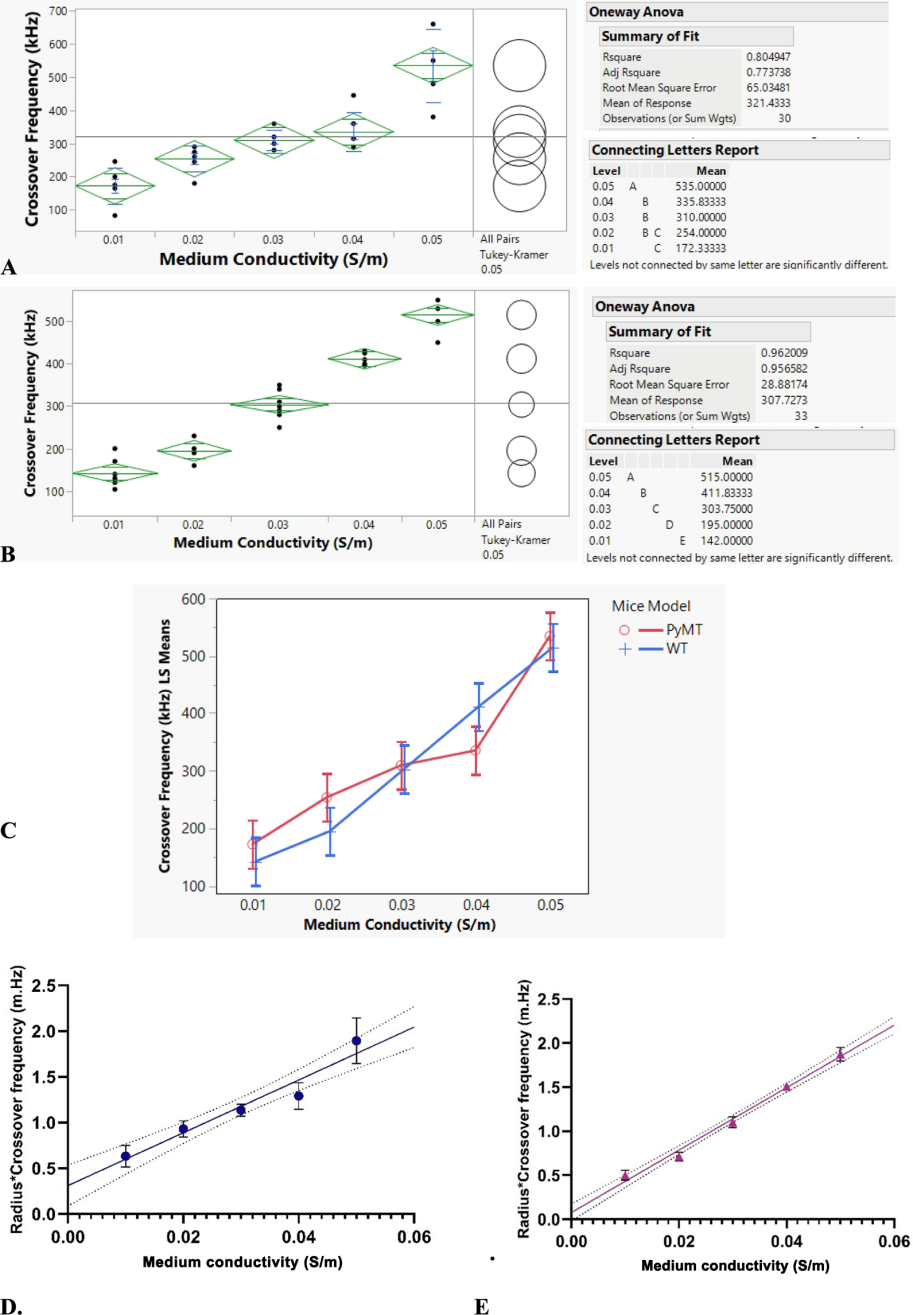
(A) One-way analysis of variance of the crossover frequency for MMTV-PyMT+ PBMCs and (B) WT PBMCs. The expression of the Polyoma middle T antigen in PyMT+ results in similar DEP responses in PBMCs, particularly at very closely matched medium conductivity values, making the medium used nearly identical. (C) The least-squares means plot examines the effects of medium conductivity levels, the mouse model factor (WT or PyMT+), and their interactions. Dielectrophoretic crossover frequency characteristics of MMTV-PyMT+ PBMCs as a function of medium conductivity are shown in parts (D) and (E). The ● points reflect the average values for MMTV-PyMT+ PBMCs, and the ▲ points reflect the average values for MMTV-WT PBMCs. The points represent the average of four measurements at each level of medium conductivity considered. The straight lines depict least-squares linear fits to the data points. Two-way Factorial Analysis showed that, on average, the PyMT+ has a higher crossover frequency than the WT.

This section presents the results of the dielectric properties obtained using peripheral blood mononuclear cells (PBMCs) derived from two groups of mice: MMTV-PyMT+ and WT. The effects of dielectrophoresis were assessed by determining the crossover frequencies of cells in the PyMT+ and WT PBMC groups. These frequencies were measured across medium conductivities, ranging from 0.01 to 0.05 S/m. The crossover frequencies across different medium conductivities were compared using an analysis of variance statistical model. The results, analyzed with JMP Pro 17.0, an advanced statistical analysis tool, revealed an insignificant overlap in the crossover frequencies between 0.01 and 0.02 S/m buffers, and also among 0.02, 0.03, and 0.04 S/m medium conductivities for MMTV-PyMT+ PBMCs, as shown by the connecting letters in the post hoc pairwise comparison test report in the ANOVA results shown in Figure 2A. In contrast, the crossover frequencies of MMTV-WT PBMCs were distinctly different, as depicted in Figure 2B. The crossover frequencies of WT PBMCs exhibit significant differences across the various medium conductivity values, with no same connected letters. From a statistical standpoint, there is insufficient evidence to conclude that the crossover frequencies differ across the considered medium conductivities, given that the p-value ≫0.05 (Figure 2A). On the other hand, for WT PBMCs, the crossover frequencies exhibit significant differences across the various medium conductivity values as indicated by all pairs of Tukey–Kramer analysis at a 95% confidence level (Figure 2B).

The results from the factorial analysis of variance, as depicted in Figure 2C, demonstrate that the conductivity of the medium significantly affects the crossover frequency in both mouse models examined. Two-way ANOVA revealed a notable interaction between the medium’s conductivity and the type of mouse model, indicating that the effect of medium conductivity on crossover frequencies varies depending on whether WT or PyMT+ PBMC is utilized.

The diameter of 28 PBMC samples was measured under the microscope (3.66 ± 0.44) μm, and the average distribution was obtained using a literature-reported value of (3.73 ± 0.36) μm from a previous study by Huang et al.^18^ This analysis was conducted using JMP Pro 17, an advanced statistical tool. The result indicates no significant difference between the calculated cell radius and the literature-reported value. Figure 2 illustrates the relationship between the cell radius, crossover frequency, and medium conductivity for MMTV-PyMT+ and WT PBMCs, using the obtained average cell radius.

Biological cells are typically homogeneous, with cytoplasm conductivity significantly higher than the plasma membrane conductivity. Consequently, the dielectric properties of biological cells can be effectively described using a single-shell model.^18^ In the case of individual PBMC cell types (PyMT+ and WT), the crossover frequency was determined at five distinct conductivities. The measurement of crossover frequency involved identifying the frequency at which the DEP force acting on the cells becomes zero, resulting in no observable movement of cells suspended in the medium when exposed to nonuniform electric field at a fixed peak-to-peak voltage (*V*_pp_). This phenomenon is dependent on the conductivity of the suspension media.

At each medium conductivity, both PyMT+ and WT PBMCs exhibited a linear relationship between crossover frequency*radius and *medium conductivity*, as depicted in Figure 2D,E. The *R-squared* values were 0.8676 and 0.9817 for PyMT+ and WT PBMCs, respectively, indicating a strong correlation. Cell dielectric properties were characterized using a single-shell model.^18,20^ The summary of the results is shown in Table 1.

**Table 1:** Estimated Dielectric Properties of MMTV-PyMT+ and WT PBMCs Using the Single-Shell Model at 0.01 ≤ σ_m_≤ 0.05 S/m Medium Conductivity^a^

	Dielectric Properties	MMTV-PyMT+ PBMCs	MMTV-WT PBMCs
1	Specific membrane capacitance, ***C*_sp-mem_** (mF/m^2^)	9.34 ± 1.12	7.60 ± 0.91
2	Membrane conductance, ***G*_mem_** (S/m^2^)	(1.89 ± 0.23) * 10^4^	(7.00 ± 0.84) * 10^3^
3	Cell interior conductivity, ***σ*_int_** (S/m)	0.069 ± 0.008	0.026 ± 0.003
4	Relative membrane permittivity,^a^***ε*_*r*-mem_**	8.44 ± 1.02	6.87 ± 0.83
5	Membrane conductivity, **σ**_**mem**_ (S/m)	(1.51 ± 0.18) * 10^–4^	(5.60 ± 0.67) * 10^–5^
6	Folding factor (φ)	1.04 ± 0.13	0.84 ± 0.10

a Where relative membrane permittivity (ε_r-mem_), a dimensionless number, is the membrane permittivity (ε_mem_) divided by the permittivity of free space, ε_o_ (8.85 × 10^–12^ F/m), i.e., ε_r-mem_ = ε_mem_/ε_o_.

**Figure 3:**
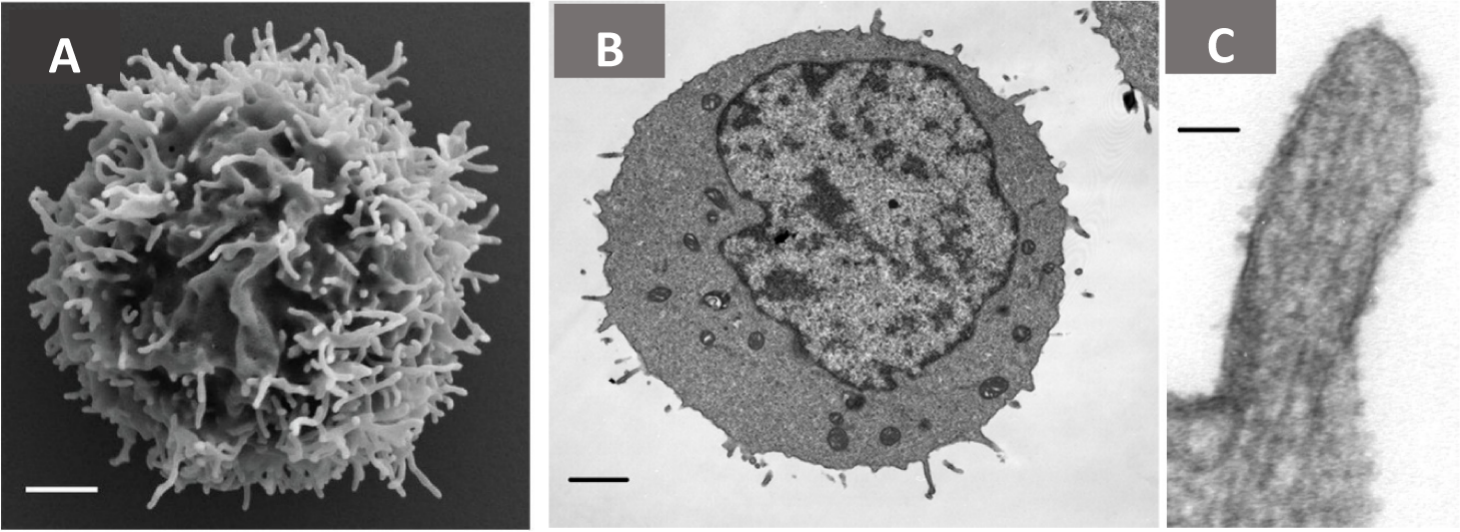
(A) Scanning electron microscopy micrograph showing microvilli that protrude from the cell surface of resting peripheral blood human T cells. Scale bar: 1 μm. Reproduced from Jung et al. with the permission of PNAS.^40^ (B) Transmission electron micrographs of an individual 300.19 cell microvillus showing that Lymphoma cell microvilli contain parallel actin filaments Scale bar: 1 μm. (C) Transmission electron microscopy micrograph showing the parallel arrangement of F-actin within the microvilli of 300.19 cells (Abelson-transformed murine pre-B lymphoma) Scale bar: 50 nm. Republished with permission of ASH and Elsevier from ref (34).

From the single-shell model analysis, it was observed that the membrane capacitance of PyMT+ PBMCs is higher than that of WT PBMCs (Table 1). The increased capacitance can be attributed to uncontrolled cell growth and division. This increased capacitance can contribute to tumor formation and the invasion of surrounding tissues. The augmented surface area of cancer cells can be attributed to their growth, expansion (protrusion), and division. Cancer cell protrusions can manifest as microvilli, filopodia, lamellipodia, and cilia.^20,31−33^ A scanning electron microscopy (SEM) analysis (Figure 3A) demonstrates microvilli protruding from the cell surface of resting peripheral blood human T cells. The transmission electron microscopy (TEM) micrograph in Figure 3B illustrates the parallel arrangement of F-actin within these microvilli. These findings were presented and explained in another study.^31−34^ Previous publications using TEM suggested that both monocytes and Polymorphonuclear Neutrophils (PMNs) possessed surface microvilli.^34^ An increase in membrane surface area or the number of ion channels can result in higher capacitance, and this could be due to increased microvillar length and reduced density, from literature.^35−37^ Microvilli increase the surface area of human blood lymphocytes appreciably (∼49%) while having little effect on cytosolic volume (∼1.6% increase), subsequently leading to an increase in surface area, resulting in higher membrane capacitance.^34^ In excitable cells, such as neurons and muscle cells, membrane capacitance is crucial in transmitting electrical signals. Changes in membrane capacitance can impact the speed and efficiency of electrical signaling in these cells.^38^ The capacitance of the cell membrane is directly proportional to the cell’s surface area and, together with membrane resistance, determines the membrane time constant. The membrane time constant governs how quickly the cell membrane potential responds to the flow of ion channel currents.^39^

Similarly, PyMT PBMCs exhibit increased membrane conductance, conductivity, and cell interior (cytoplasm) conductivity compared to those in the WT PBMC model. These heightened properties are attributable to the cellular changes induced by the overexpression in the PyMT model and could be attributed to breast cancer in women. In late-stage primary tumors and lung metastases from MMTV-PyMT+ mice, an androgen receptor (AR) protein expression mechanistic study revealed that abundant nuclear AR protein promotes tumor survival,^41^ thus increasing proliferation, migration, invasion, and anchorage-independent growth. These findings indicate that the bioelectric characteristics of cancerous tissue differ from those of normal tissue and may evolve during cancer development.^42^ As we investigate the reasons for these characteristic changes in the PBMCs from PyMT+ mice, our previous work demonstrated that defects in the cell membrane can alter its electrical properties, potentially leading to increased conductivity.^19,26,27,43,44^ However, it is essential to note that membrane conductivity is lower when the cell is intact and healthy compared to when the membrane is damaged or defective, in the case of an infection or abnormality in the cell (observed from our previous research studies).^19,26,27,43,44^

Another property of interest in this study is the membrane folding factor.^26^ Using the single-shell model, the dielectric properties of MMTV-PyMT+ and WT were estimated, and it was revealed that PyMT+ PBMCs have a higher folding factor than WT. The folding factor describes the roughness or topography of the cell’s plasma membrane surface.^11,45,46^ This parameter can be expressed as the ratio of the actual membrane capacitance to that of a smooth membrane (φ= *C*_actual_/*C*_smooth_),^47,48^ with the assumption that the smooth membrane has a specific capacitance of 9.0 mF/m^2^,^26^ and sometimes reported as 6.0 mF/m^2^ or 8.0 mF/m^2^.^45^ For this work, we considered smooth membrane surface capacitance to be 9.0 mF/m^2^. WT PBMCs have a lower membrane folding factor, which can be readily attributed to their increased microvilli length, as suggested in some previous studies,^35,36^ as a result of the formation of short, branched filaments that mediate the formation of long, parallel actin filaments found in microvilli. Some contributing factors to increased membrane folding in PyMT+ PBMCs are protein misfolding and aggregation,^49^ cell signaling, and transport of ions across the membrane, which is critical for the proper functioning of cells. Overexpression of folding factors may lead to increased protein folding, and overexpression of proteins with strong demands for those resources might cause resource overloads,^50,51^ which is abnormal and can lead to cellular defects.^50^ The process of polytopic (multispanning) membrane protein folding can be understood as a series of sequential but potentially overlapping steps, including (i) formation, orientation, and integration of transmembrane helices in the lipid bilayer, (ii) helical packing within the membrane, (iii) localization and folding of cytosolic and extracytoplasmic domains, and (iv) for many proteins, quaternary organization into functional oligomers. The complex cell surface morphology, characterized by abundant membrane folds, microvilli, filopodia, and other membrane extensions, is believed to contribute to the highly invasive behavior and therapy resistance.^47^

### 4.1 Impact of Frequency on DEP

**Figure 4:**
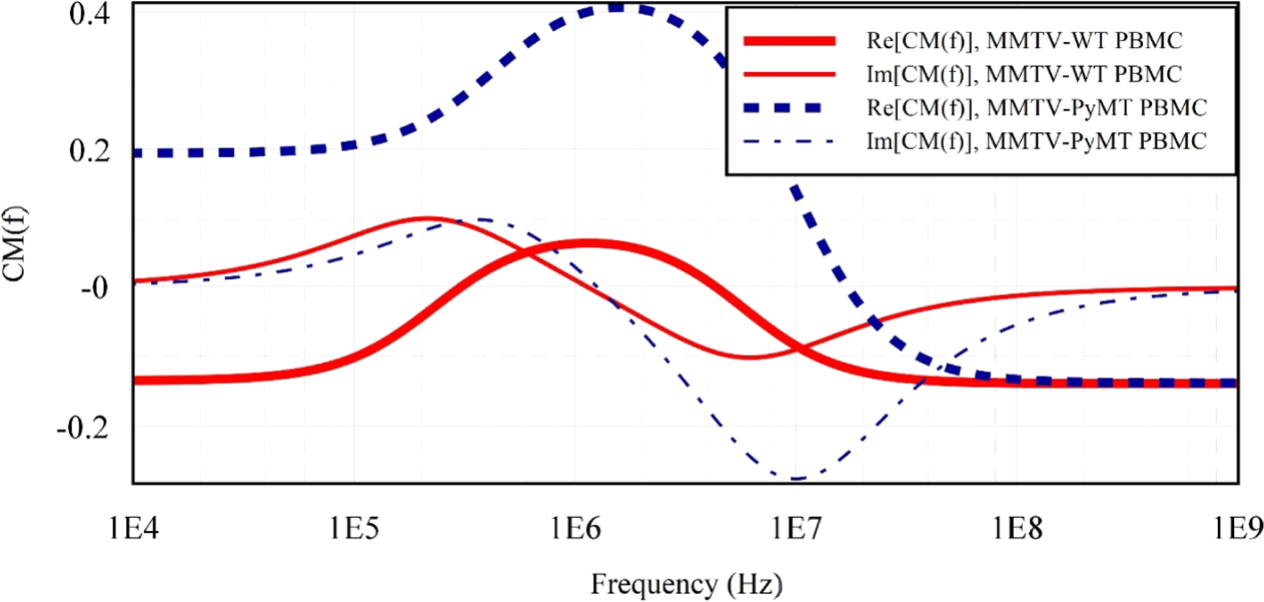
Real and Imaginary plot of the Clausius–Mossotti factor, (CM) for MMTV-PyMT+ and MMTV-WT PBMCs as a function of frequency using the single-shell model calculated data in Table 1 at 0.02 S/m medium conductivity. PyMT+ has a greater ability to polarize and interact more with the electric field. PyMT+ PBMCs experiences more energy loss, as indicated by its higher Im(CM).

The Clausius–Mossotti (CM) factor is an important parameter that describes the polarization of a bioparticle suspended in a medium with a set of dielectric properties different from that of the particle itself. In this study, MATLAB is used to simulate the real part of the Clausius-Mossotti [Re(CM)] factor across a suitable frequency range at a fixed *V*_pp_ to predict the crossover frequency (*see*Figure 4), and MyDEP software^52^ was to simulate both the real and imaginary parts of the CM factor, which was also used in a similar study.^52^ [Re (CM)] of PyMT+ PBMCs in Figure 4 is higher, indicating a greater ability to polarize and interact with the electric field due to its higher permittivity and conductivity, enabling a stronger interaction. This could be due to the expression of the Polyoma middle T antigen. The middle T antigen (MT) is carried by a virus and is defective for transformation *in vitro* or tumorigenesis. The details of this defectiveness have been explained in another study.^53−57^ The irregular shape developed by the immune cells and an increased volume have great potential to account for the higher polarizability of the PyMT+ PBMCs. This attribute aligns with a study of the polarizability of macromolecules for single-molecule optical biosensing.^58^ The imaginary part of the CM factor, Im(CM), is associated with the absorption and dissipation of energy by particles during dielectrophoresis. The imaginary part also relates to the particle’s dielectric loss or electrical conductivity. It contributes to the rotational movement of the particle under the influence of the electric field. PyMT+ PBMCs have a higher dielectric energy loss compared to WT. However, the energy is dissipated in an oscillatory manner and not as heat because the imaginary CM factor spectrum was more in the negative axis region, when the cell exhibits a capacitive response to the applied nonuniform electric field, causing the PyMT+ cells to accumulate electrical charge on its surfaces, and creating an electric field within itself, because of the larger surface area, and higher membrane permittivity, as demonstrated by Larsen et al.^59^

**Figure 5:**
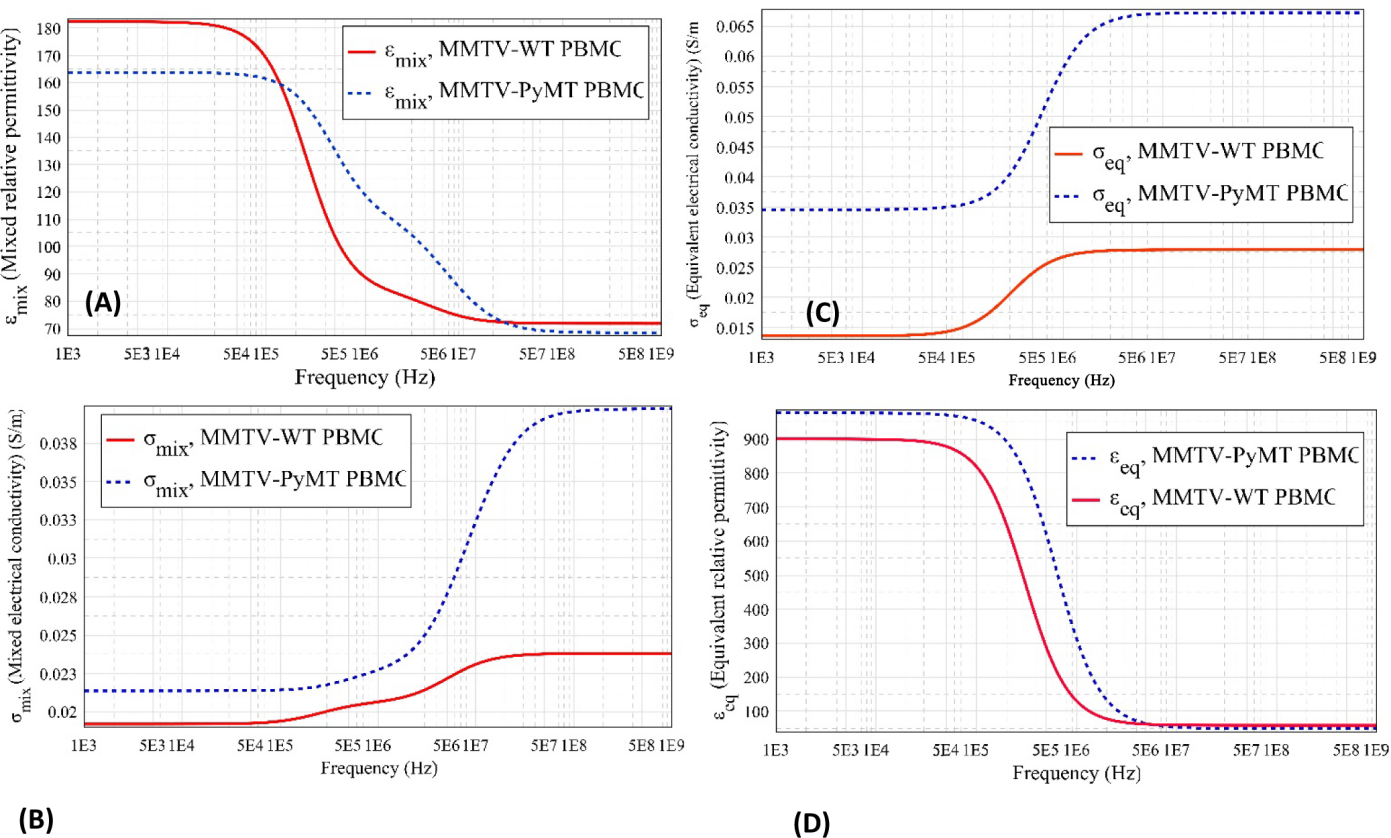
Plots illustrate the changes in the dielectric response of MMTV-PyMT+ and WT PBMCs in a suspension with respect to frequency variation, using the single-shell model calculated data from Table 1 at a medium conductivity of 0.02 S/m. (A) Mixture dielectric constant (*ε*_mix_), (B) equivalent dielectric (ε_eq_), (C) mixture conductivity, σ_mix_, and (D) equivalent electrical conductivity (σ_eq_) are shown. The dielectric response to the electric field without the contribution of the cell particles is represented by (σ_eq_) and (ε_eq_), while the dielectric response with the contribution of the cell particles is denoted by (σ_mix_) and (ε_mix_).

Generally, the equivalent electrical conductivity (*σ*_eq_) refers to the conductivity of a solution containing multiple ions. It represents the overall electrical conductivity resulting from the combined effect of all the ions in the suspending media without considering the bioparticles. Each ion contributes to the overall electrical conductivity based on its conductivity and concentration. Therefore, it is consistent to say equivalent electrical conductivity is determined by the cumulative contributions of all the ions in the suspension. Similarly, the mixed conductivity (*σ*_mix_) of the suspension is related to the combined ionic and electric conductivity observed in the PyMT+ PBMC cell membrane when suspended in the medium. This study uses the dielectrophoresis technique to investigate the interaction between electric pulses and MMTV-PyMT+ and WT PBMCs. Understanding the interaction between electric pulses and cells have various biomedical applications.^60^Figure 5 illustrates how the equivalent and mixture conductivities of the suspension change over the frequency range. The increased equivalent conductivity of the PyMT+ suspension is attributed to the structural changes experienced by the cells due to the expression of the Polyoma middle T antigen. In general, the changes in effective conductivity were analyzed in a study by Pavlin and Miklavčič for different parameters, including cell volume fraction, membrane and medium conductivity, critical transmembrane potential, and cell orientation.^60^ In many DEP applications, mixed conductivity and permittivity are preferred, because they accurately represent the complex interactions within the heterogeneous medium, providing more realistic modeling and cell analysis.

When an electric field is applied to the cell suspension, it induces an inhomogeneous transmembrane potential (IMP), also known as a nonuniform transmembrane potential (TMP). If the IMP exceeds its critical value, TMP_c_,^61^ the cell membrane undergoes reversible permeabilization, referred to as electro-permeabilization, allowing the transport of ions and molecules into the cell membrane.^61−65^ Theories explaining the dielectric and conductive properties of cell suspensions, such as the Maxwell eqn., help understand the experiments conducted at a microscopic level, such as in a microfluidic device, to gain knowledge about biophysical processes on a macroscopic level. The increased equivalent conductivity of the PyMT+ PBMCs suspension is attributed to the diffusion of ions from the cells, which is consistent with the suspended cells’ behavior.^66^
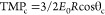
3

where θ_c_ denotes the critical angle, *R* is the radius of the cell membrane, and *E*_0_ represents the electric field.

Equivalent relative permittivity results from the contribution of both the suspending medium and the suspended cells under the influence of an electric field (Figure 5A). The DEP force, which arises from the polarizability of the cells, is dependent on the permittivity of the medium. Figure 5C demonstrates that PyMT+ PBMCs have a increased equivalent permittivity compared to that of WT PBMCs. This is supported by the results reported in Table 1, where PyMT+ exhibits a higher permittivity value. This higher permittivity allows for quicker penetration of the frequency, resulting in lower frequency dispersion. This phenomenon arises from counterion conduction and fluctuations near the charged surface groups on the cell membrane. In other words, the frequency penetrates the cell membrane of MMTV-PyMT+ faster than it does in WT PBMCs. MyDEP program utilizes the Maxwell–Wagner model and the Clausius–Mossotti factor (eq 2) to compute the permittivity. In Figure 5A,C, it is important to note that the steep fall and rise in permittivity, respectively, as the frequency increases is attributed to the electrode polarization phenomenon, addressed in another study.^67^ As the cell membrane becomes more conductive, the effective penetration depth of the electric field increases.

**Figure 6:**
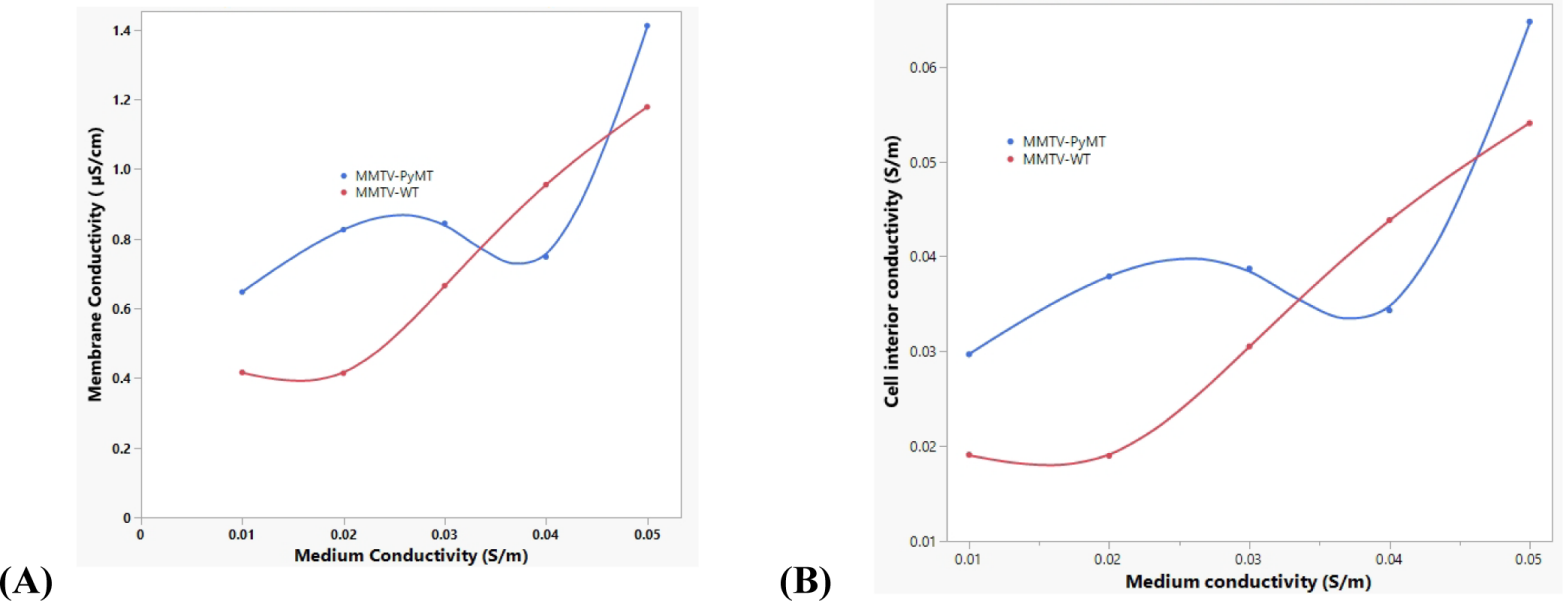
Correlation between the conductivity of the cell plasma membrane and medium conductivity, as shown in (A), and the conductivity of the cell interior and medium conductivity, as shown in (B). Given the estimated R-squared value of 0.59 for the simple linear regression, the plots show how overexpression in PyMT+ mice causes abnormalities in the cell membrane potential (*V*_m_), resulting in nonlinearity between the membrane and intracellular conductivity plotted on the *y*-axis at varying medium conductivity on the *x*-axis. Wild type, on the other hand, has an R-squared value of 0.94, indicating a strong linear relationship between the response and predictor variables. This observation therefore discriminates PyMT+ from WT.

The results shown in Figure 6 indicate that the conductivity of the cytoplasm and cell membrane shows a linear relationship with an R-squared value of approximately 0.94 for WT PBMCs, while it is 0.59 for PyMT+ PBMCs. This indicates that medium conductivity accounts for 94% of the variation in the membrane and intracellular conductivity in WT PBMCs, whereas medium conductivity accounts for only 59% of the variation in the membrane and intracellular conductivity in PyMT+ PBMCs. Various ion channels and transporters at the cell plasma membrane provide different permeability to distinct ions like Na^+^, K^+^, Ca^2+^, and Cl^–^. Unequal distribution of these ions results in membrane potential (V_m_).^68^ Overexpression of the PyMT+ mice could be the cause of the abnormality or ion channel dysfunction in the cell membrane potential (V_m_) leading to a nonlinear effect on cell interior conductivity. Lower cell interior conductivity can be a result of K^+^ efflux from the intracellular environment through the K^+^ channel, making numbers of K^+^ ions unstable in the cell, as seen in RBCs and cancer cells.^68,69^ On the other hand, K^+^ has been well-established to be stable in noncancerous tissues,^68^ owing to its tightly regulated movement in normal cell physiological conditions.

## 5 Conclusions

The dielectrophoresis technique was employed for the first time to characterize and analyze the dielectric properties of MMTV-PyMT+ and wild-type PBMCs. Literature supports the use of the single-shell model for accurately simulating experimental data below 1 MHz. From the results obtained, the Polyoma middle T antigen expression in mice at 14+ weeks alters the dielectric properties of PBMCs compared to the WT PBMCs, which serves as our control. From the results obtained, it is reasonable to anticipate that PBMCs from breast cancer patients will provide a distinct and unique DEP response compared to that of normal PBMCs. PyMT+ PBMCs show increased polarizability, membrane capacitance, a higher folding factor, and disrupted membrane potential, leading to unstable cell interior conductivity.

The MyDEP tool is used to simulate the cell suspension’s dielectric constant and conductivity thus yielding dispersion curves. The increased conductance in PyMT+ PBMCs can be attributed to transmembrane conductance, apparent ion outflow from the cytoplasm, or electrotransfer during the experiment, all of which depend on the characteristics of the medium^70^ and the extent of interaction with the electric field. This explains the higher equivalent conductivity and permittivity observed in the PyMT+ PBMCs, which can be attributed to the same reasons. The CM curves demonstrate a potential separation frequency range of 150 kHz to 10–15 MHz. These results suggest that a low to medium conductivity range is more suitable for diagnosing stage IV breast cancer utilizing dielectrophoresis.

Based on these findings, the DEP technique effectively characterizes late-stage breast cancer and could be translatable to humans. This shows that the estimated conductance and dielectric constant can be utilized to understand electrotransfer in the cell, which has significant applications in drug delivery and the transfer of ions and molecules to the cell membrane. Overall, this study provides a compelling alternative approach for estimating the dielectric properties of late-stage breast cancer using PBMCs from the MMTV-PyMT mouse model. These data (dielectric properties) can aid in developing cancer biosensors and detection devices.

## Data Availability

The data supporting this study’s findings are mentioned within the manuscript in the results and discussion section. No other data were generated beyond what is provided in this manuscript.

## References

[ref1] Siegel R. L.; Miller K. D.; Wagle N. S.; Jemal A. Cancer statistics. Ca-Cancer J. Clin. 2023, 73 (1), 17–48. 10.3322/caac.21763. 36633525

[ref2] Amarante M. K.; de Sousa Pereira N.; Vitiello G. A. F.; Watanabe M. A. E. Involvement of a mouse mammary tumor virus (MMTV) homologue in human breast cancer: Evidence for, against and possible causes of controversies. Microb. Pathog. 2019, 130, 283–294. 10.1016/j.micpath.2019.03.021. 30905715

[ref3] Rennhack J. P.; To B.; Swiatnicki M.; Dulak C.; Ogrodzinski M. P.; Zhang Y.; Li C.; Bylett E.; Ross C.; Szczepanek K.; Hanrahan W.; Jayatissa M.; Lunt S. Y.; Hunter K.; Andrechek E. R. Integrated analyses of murine breast cancer models reveal critical parallels with human disease. Nat. Commun. 2019, 10 (1), 3261 10.1038/s41467-019-11236-3. 31332182 PMC6646342

[ref4] Regua A. T.; Arrigo A.; Doheny D.; Wong G. L.; Lo H.-W. Transgenic mouse models of breast cancer. Cancer Lett. 2021, 516, 73–83. 10.1016/j.canlet.2021.05.027. 34090924 PMC8260455

[ref5] Attalla S.; Taifour T.; Bui T.; Muller W. Insights from transgenic mouse models of PyMT-induced breast cancer: Recapitulating human breast cancer progression in vivo. Oncogene 2021, 40 (3), 475–491. 10.1038/s41388-020-01560-0. 33235291 PMC7819848

[ref6] Gross E. T.; Han S.; Vemu P.; Peinado C. D.; Marsala M.; Ellies L. G.; Bui J. D. Immunosurveillance and immunoediting in MMTV-PyMT-induced mammary oncogenesis. Oncoimmunology 2017, 6 (2), e1268310 10.1080/2162402X.2016.1268310. 28344881 PMC5353915

[ref7] Lin E. Y.; Jones J. G.; Li P.; Zhu L.; Whitney K. D.; Muller W. J.; Pollard J. W. Progression to malignancy in the polyoma middle T oncoprotein mouse breast cancer model provides a reliable model for human diseases. Am. J. Pathol. 2003, 163, 2113–2126. 10.1016/S0002-9440(10)63568-7. 14578209 PMC1892434

[ref8] Jiang G.; Tu J.; Zhou L.; Dong M.; Fan J.; Chang Z.; Zhang L.; Bian X.; Liu S. Single-cell transcriptomics reveal the heterogeneity and dynamic of cancer stem-like cells during breast tumor progression. Cell Death Dis. 2021, 12 (11), 979 10.1038/s41419-021-04261-y. 34675206 PMC8531288

[ref9] Guy C. T.; Cardiff R. D.; Muller W. J. Induction of Mammary Tumors by Expression of Polyomavirus Middle T Oncogene: A Transgenic Mouse Model for Metastatic Disease. Mol. Cell. Biol. 1992, 12 (3), 954–961. 10.1128/mcb.12.3.954-961.1992. 1312220 PMC369527

[ref10] Goodman L. J.; Kain S. R.; Firestone G. L. Trafficking of wild-type and an endoproteolytic-site mutant of the mouse mammary tumor virus glycoprotein. J. Biol. Chem. 1993, 268 (4), 2329–2336. 10.1016/S0021-9258(18)53779-0. 8381404

[ref11] Pohl H. A. The Motion and Precipitation of Suspensoids in Divergent Electric Fields. J. Appl. Phys. 1951, 22 (7), 869–871. 10.1063/1.1700065.

[ref12] Küttel C.; Nascimento E.; Demierre N.; Silva T.; Braschler T.; Renaud P.; Oliva A. G. Label-free detection of Babesia bovis infected red blood cells using impedance spectroscopy on a microfabricated flow cytometer. Acta Trop. 2007, 102, 63–68. 10.1016/j.actatropica.2007.03.002. 17451631

[ref13] Schwan H. P. Electrical properties of tissue and cell suspensions. Adv. Biol. Med. Phys. 1957, 5, 147–209. 10.1016/B978-1-4832-3111-2.50008-0. 13520431

[ref14] Michael K. A.; Hiibel S. R.; Geiger E. J. Dependence of the dielectrophoretic upper crossover frequency on the lipid content of microalgal cells. Algal Res. 2014, 6, 17–21. 10.1016/j.algal.2014.08.004.

[ref15] Hughes M. P. Strategies for dielectrophoretic separation in laboratory-on-a-chip systems. Electrophoresis 2002, 23 (16), 2569–2582. 10.1002/1522-2683(200208)23:16<2569::AID-ELPS2569>3.0.CO;2-M. 12210160

[ref16] Hoettges K. F.; Hübner Y.; Broche L. M.; Ogin S. L.; Kass G. E. N.; Hughes M. P. Dielectrophoresis-activated multiwell plate for label-free high-throughput drug assessment. Anal. Chem. 2008, 80, 2063–2068. 10.1021/ac702083g. 18278948

[ref17] Wang L.; Lu J.; Marchenko S. A.; Monuki E. S.; Flanagan L. A.; Lee A. P. Dual frequency dielectrophoresis with interdigitated sidewall electrodes for microfluidic flow-through separation of beads and cells. Electrophoresis 2009, 30, 782–791. 10.1002/elps.200800637. 19197906

[ref18] Huang Y.; Joo S.; Duhon M.; Heller M.; Wallace B.; Xu X. Dielectrophoretic cell separation and gene expression profiling on microelectronic chip arrays. Anal. Chem. 2002, 74 (14), 3362–3371. 10.1021/ac011273v. 12139041

[ref19] Oladokun R.; Adekanmbi E. O.; An V.; Gangavaram I.; Srivastava S. K. Dielectrophoretic profiling of erythrocytes to study the impacts of metabolic stress, temperature, and storage duration utilizing a point-and-planar microdevice. Sci. Rep. 2023, 13 (1), 17281 10.1038/s41598-023-44022-9. 37828082 PMC10570315

[ref20] Huang Y.; Holzel R.; Pethig R.; Wang X.-B. Differences in the AC electrodynamics of viable and non-viable yeast cells determined through combined dielectrophoresis and electrorotation studies. Phys. Med. Biol. 1992, 37 (7), 1499 10.1088/0031-9155/37/7/003. 1631195

[ref21] Schwan H. P.; Takashima S.; Miyamoto V. K.; Stoeckenius W. Electrical Properties of Phospholipid Vesicles. Biophys. J. 1970, 10 (11), 1102–1119. 10.1016/S0006-3495(70)86356-1. 5471701 PMC1367986

[ref22] Foster K. R.; Sauer F. A.; Schwan H. P. Electrorotation and levitation of cells and colloidal particles. Biophys. J. 1992, 63 (1), 180–190. 10.1016/S0006-3495(92)81588-6. 19431839 PMC1262135

[ref23] Giduthuri A. T.; Adekanmbi E. O.; Srivastava S. K.; Moberly J. G. Dielectrophoretic ultra-high-frequency characterization and in silico sorting on uptake of rare earth elements by Cupriavidus necator. Electrophoresis 2021, 42 (5), 656–666. 10.1002/elps.202000095. 33215725

[ref24] Adekanmbi E. O.; Giduthuri A. T.; Waymire S.; Srivastava S. K. Utilization of Dielectrophoresis for the Quantification of Rare Earth Elements Adsorbed on Cupriavidus necator. ACS Sustainable Chem. Eng. 2020, 8 (3), 1353–1361. 10.1021/acssuschemeng.9b03878.

[ref25] Pethig R.; Jakubek L. M.; Sanger R. H.; Heart E.; Corson E. D.; Smith P. J. Electrokinetic measurements of membrane capacitance and conductance for pancreatic beta-cells. lIEE Proc.: Nanobiotechnol. 2005, 152 (6), 189–193. 10.1049/ip-nbt:20050040. 16441179

[ref26] Oladokun R.; Adekanmbi E.; Ueti M.; Srivastava S. K. Dielectric characterization of Babesia bovis using the dielectrophoretic crossover frequency. Electrophoresis 2023, 44, 988–1001. 10.1002/elps.202200263. 37160713

[ref27] Giduthuri A. T.; Theodossiou S. K.; Schiele N. R.; Srivastava S. K. Dielectrophoretic Characterization of Tenogenically Differentiating Mesenchymal Stem Cells. Biosensors 2021, 11, 50 10.3390/bios11020050. 33669223 PMC7919818

[ref28] Oladokun R.; Smith C.; Eubank T.; Srivastava S. K.;Dielectrophoresis-based breast cancer study: Characterization and separation of peripheral blood mononuclear cells from PyMT and WT mouse model In 2023 AIChE Annual Meeting, AIChE, 2023.

[ref29] Oladokun R.; Srivastava S. K.; Eubank T.;Dielectrophoresis-based detection of breast cancer using peripheral blood mononuclear cells in a ductal adenocarcinoma PyMT± mouse model on a microfluidic device In 2023 AIChE Annual Meeting, AIChE, 2023.

[ref30] Srivastava S. K.; Giduthuri A. T. Microfluidic-Chip Technology for Disease Diagnostic Applications via Dielectrophoresis. Nanosensors for Futuristic Smart and Intelligent Healthcare Systems 2022, 318 10.1201/9781003093534-17.

[ref31] Svitkina T. M.; Bulanova E. A.; Chaga O. Y.; Vignjevic D. M.; Kojima S.-I.; Vasiliev J. M.; Borisy G. G. Mechanism of filopodia initiation by reorganization of a dendritic network. J. Cell Biol. 2003, 160, 409–421. 10.1083/jcb.200210174. 12566431 PMC2172658

[ref32] Jacquemet G.; Hamidi H.; Ivaska J. Filopodia in cell adhesion, 3D migration and cancer cell invasion. Curr. Opin. Cell Biol. 2015, 36, 23–31. 10.1016/j.ceb.2015.06.007. 26186729

[ref33] Arjonen A.; Kaukonen R.; Ivaska J. Filopodia and adhesion in cancer cell motility. Cell Adhes. Migr. 2011, 5 (5), 421–430. 10.4161/cam.5.5.17723. PMC3218609 21975551

[ref34] Majstoravich S.; Zhang J.; Nicholson-Dykstra S.; Linder S.; Friedrich W.; Siminovitch K. A.; Higgs H. N. Lymphocyte microvilli are dynamic, actin-dependent structures that do not require Wiskott-Aldrich syndrome protein (WASp) for their morphology. Blood 2004, 104 (5), 1396–1403. 10.1182/blood-2004-02-0437. 15130947

[ref35] Shelley C. S.; Remold-O’Donnell E.; Davis A. E. III; Bruns G. A.; Rosen F. S.; Carroll M. C.; Whitehead A. S. Molecular characterization of sialophorin (CD43), the lymphocyte surface sialoglycoprotein defective in Wiskott-Aldrich syndrome. Proc. Int. Acad. Sci. 1989, 86, 2819–2823. 10.1073/pnas.86.8.2819. PMC287010 2784859

[ref36] Candotti F.; Facchetti F.; Blanzuoli L.; Stewart D. M.; Nelson D. L.; Blaese R. M. Retrovirus-mediated WASP gene transfer corrects defective actin polymerization in B cell lines from Wiskott–Aldrich syndrome patients carrying ‘null’ mutations. Gene Ther. 1999, 6 (6), 1170–1174. 10.1038/sj.gt.3300926. 10455421

[ref37] Molina I. J.; Kenney D. M.; Rosen F. S.; Remold-O’Donnell E. T cell lines characterize events in the pathogenesis of the Wiskott-Aldrich syndrome. J. Exp. Med. 1992, 176 (3), 867–874. 10.1084/jem.176.3.867. 1512549 PMC2119357

[ref38] Betts J. G. Anatomy and Physiology; OpenStax College, Rice University, 2013.

[ref39] Golowasch J.; Nadim F. Capacitance, Membrane. In Computational Neuroscience, Jaeger D.; Jung R., Eds.; Springer: New York, 2013; pp. 1 5.

[ref40] Jung Y.; Riven I.; Feigelson S. W.; Kartvelishvily E.; Tohya K.; Miyasaka M.; Alon R.; Haran G. Three-dimensional localization of T-cell receptors in relation to microvilli using a combination of superresolution microscopies. Proc. Natl. Acad. Sci. 2016, 113, E5916–E5924. 10.1073/pnas.1605399113. 27647916 PMC5056101

[ref41] Christenson J. L.; Butterfield K. T.; Spoelstra N. S.; Norris J. D.; Josan J. S.; Pollock J. A.; McDonnell D. P.; Katzenellenbogen B. S.; Katzenellenbogen J. A.; Richer J. K. MMTV-PyMT and Derived Met-1 Mouse Mammary Tumor Cells as Models for Studying the Role of the Androgen Receptor in Triple-Negative Breast Cancer Progression. Horm. Cancer 2017, 8 (2), 69–77. 10.1007/s12672-017-0285-6. 28194662 PMC5407486

[ref42] Zhu K.; Hum N. R.; Reid B.; Sun Q.; Loots G. G.; Zhao M. Electric Fields at Breast Cancer and Cancer Cell Collective Galvanotaxis. Sci. Rep. 2020, 10 (1), 8712 10.1038/s41598-020-65566-0. 32457381 PMC7250931

[ref43] Chiok K. L.; Paul N. C.; Adekanmbi E. O.; Srivastava S. K.; Shah D. H. Dimethyl adenosine transferase (KsgA) contributes to cell-envelope fitness in *Salmonella* Enteritidis. Microbiol. Res. 2018, 216, 108–119. 10.1016/j.micres.2018.08.009. 30269850 PMC6628923

[ref44] Giduthuri A. T.; Theodossiou S. K.; Schiele N. R.; Srivastava S. K. Dielectrophoretic Characterization of Tenogenically Differentiating Mesenchymal Stem Cells. Biosensors 2021, 11 (2), 50 10.3390/bios11020050. 33669223 PMC7919818

[ref45] Pethig R. R. Dielectrophoresis: Theory, Methodology, and Biological Applications, 1st ed.; John Wiley & Sons, Ltd: United Kingdom, 2017; pp 428.

[ref46] Persing D. H.; Mathiesen D.; Marshall W. F.; Telford S. R.; Spielman A.; Thomford J. W.; Conrad P. A. Detection of Babesia microti by polymerase chain reaction. J. Clin. Microbiol. 1992, 30 (8), 2097–2103. 10.1128/jcm.30.8.2097-2103.1992. 1500517 PMC265450

[ref47] Memmel S.; Sukhorukov V. L.; Höring M.; Westerling K.; Fiedler V.; Katzer A.; Krohne G.; Flentje M.; Djuzenova C. S. Cell surface area and membrane folding in glioblastoma cell lines differing in PTEN and p53 status. PLoS One 2014, 9, e87052 10.1371/journal.pone.0087052. 24498019 PMC3909012

[ref48] Gascoyne P. R.; Shim S.; Noshari J.; Becker F. F.; Stemke-Hale K. Correlations between the dielectric properties and exterior morphology of cells revealed by dielectrophoretic field-flow fractionation. Electrophoresis 2013, 34 (7), 1042–1050. 10.1002/elps.201200496. 23172680 PMC3754903

[ref49] Vabulas R. M.; Raychaudhuri S.; Hayer-Hartl M.; Hartl F. U. Protein folding in the cytoplasm and the heat shock response. Cold Spring Harbor Perspect. Biol. 2010, 2, a004390 10.1101/cshperspect.a004390. PMC2982175 21123396

[ref50] Moriya H. Quantitative nature of overexpression experiments. Mol. Biol. Cell 2015, 26 (22), 3932–3939. 10.1091/mbc.E15-07-0512. 26543202 PMC4710226

[ref51] Snoep J.; Yomano L.; Westerhoff H.; Ingram L. Protein burden in Zymomonas mobilis: Negative flux and growth control due to overproduction of glycolytic enzymes. Microbiology 1995, 141, 2329–2337. 10.1099/13500872-141-9-2329.

[ref52] Cottet J.; Fabregue O.; Berger C.; Buret F.; Renaud P.; Frénéa-Robin M. MyDEP: A New Computational Tool for Dielectric Modeling of Particles and Cells. Biophys. J. 2019, 116 (1), 12–18. 10.1016/j.bpj.2018.11.021. 30558882 PMC6342686

[ref53] Bronson R.; Dawe C.; Carroll J.; Benjamin T. Tumor induction by a transformation-defective polyoma virus mutant blocked in signaling through Shc. Proc. Int. Acad. Sci. 1997, 94, 7954–7958. 10.1073/pnas.94.15.7954. PMC21536 9223294

[ref54] Culleré X.; Rose P.; Thathamangalam U.; Chatterjee A.; Mullane K. P.; Pallas D. C.; Benjamin T. L.; Roberts T. M.; Schaffhausen B. S. Serine 257 Phosphorylation Regulates Association of Polyomavirus Middle T Antigen with 14-3-3 Proteins. J. Virol. 1998, 72, 558–563. 10.1128/jvi.72.1.558-563.1998. 9420259 PMC109408

[ref55] Freund R.; Sotnikov A.; Bronson R. T.; Benjamin T. L. Polyoma virus middle T is essential for virus replication and persistence as well as for tumor induction in mice. Virology 1992, 191, 716–723. 10.1016/0042-6822(92)90247-M. 1333120

[ref56] Yin J. J.; Selander K.; Chirgwin J. M.; Dallas M.; Grubbs B. G.; Wieser R.; Massagué J.; Mundy G. R.; Guise T. A. TGF-beta signaling blockade inhibits PTHrP secretion by breast cancer cells and bone metastases development. J. Clin. Invest. 1999, 103, 197–206. 10.1172/JCI3523. 9916131 PMC407876

[ref57] Whalen K. A.; Weber G. F.; Benjamin T. L.; Schaffhausen B. S. Polyomavirus middle T antigen induces the transcription of osteopontin, a gene important for the migration of transformed cells. J. Virol. 2008, 82, 4946–4954. 10.1128/jvi.02650-07. 18337582 PMC2346735

[ref58] Booth L. S.; Browne E. V.; Mauranyapin N. P.; Madsen L. S.; Barfoot S.; Mark A.; Bowen W. P. Modelling of the dynamic polarizability of macromolecules for single-molecule optical biosensing. Sci. Rep. 2022, 12 (1), 1995 10.1038/s41598-022-05586-0. 35132077 PMC8821610

[ref59] Larsen D.; Cox K.; Reusser D., Polarizability. California State University Affordable Learning Solutions Program. https://chem.libretexts.org/Bookshelves/Physical_and_Theoretical_Chemistry_Textbook_Maps/Supplemental_Modules_(Physical_and_Theoretical_Chemistry)/Physical_Properties_of_Matter/Atomic_and_Molecular_Properties/Intermolecular_Forces/Specific_Interactions/Polarizability. (Accessed 21 10 2023).

[ref60] Pavlin M.; Miklavčič D. Effective Conductivity of a Suspension of Permeabilized Cells: A Theoretical Analysis. Biophys. J. 2003, 85 (2), 719–729. 10.1016/S0006-3495(03)74515-9. 12885623 PMC1303197

[ref61] Gabriel B.; Teissié J. Fluorescence imaging in the millisecond time range of membrane electropermeabilisation of single cells using a rapid ultra-low-light intensifying detection system. Eur. Biophys. J. 1998, 27 (3), 291–298. 10.1007/s002490050136.

[ref62] Neumann E.; Schaefer-Ridder M.; Wang Y.; Hofschneider P. H. Gene transfer into mouse lyoma cells by electroporation in high electric fields. EMBO J. 1982, 1 (7), 841–845. 10.1002/j.1460-2075.1982.tb01257.x. 6329708 PMC553119

[ref63] Zimmermann U. Electric field-mediated fusion and related electrical phenomena. Biochim. Biophys. Acta 1982, 694 (3), 227–277. 10.1016/0304-4157(82)90007-7. 6758848

[ref64] Weaver J. C.; Chizmadzhev Y. A. Theory of electroporation: A review. Bioelectrochem. Bioenerg. 1996, 41 (2), 135–160. 10.1016/S0302-4598(96)05062-3.

[ref65] Tsong T. Y. Electroporation of cell membranes. Biophys J. 1991, 60 (2), 297–306. 10.1016/S0006-3495(91)82054-9. 1912274 PMC1260065

[ref66] Cooper G. M. The Cell: A Molecular Approach, 2nd ed.; Sinauer Associates: Sunderland, MA, 2000.

[ref67] Raicu V.; Kitagawa N.; Irimajiri A. A quantitative approach to the dielectric properties of the skin. Phys. Med. Biol. 2000, 45 (2), L1 10.1088/0031-9155/45/2/101. 10701501

[ref68] Yang M.; Brackenbury W. J. Membrane potential and cancer progression. Front. Physiol. 2013, 4, 185 10.3389/fphys.2013.00185. 23882223 PMC3713347

[ref69] von Lindern M.; Egée S.; Bianchi P.; Kaestner L. The Function of Ion Channels and Membrane Potential in Red Blood Cells: Toward a Systematic Analysis of the Erythroid Channelome. Front. Physiol. 2022, 13, 824478 10.3389/fphys.2022.824478. 35177994 PMC8844196

[ref70] Adekanmbi E. O.; Dustin J.; Srivastava S. K. Electro-osmotic surface effects generation in an electrokinetic-based transport device: A comparison of RF and MW plasma generating sources. Electrophoresis 2019, 40, 1573–1579. 10.1002/elps.201800464. 30762241

